# MAFF as a potential novel protective factor against Parkinson’s disease: evidence from Mendelian randomization and single-cell RNA sequencing

**DOI:** 10.3389/fnagi.2026.1759121

**Published:** 2026-09-11

**Authors:** Xinyao Li, Qiuyan Wang, Guowen Min, Xiaoli Yuan, Yuling Tian

**Affiliations:** Department of Neurology, The First Hospital of Shanxi Medical University, Taiyuan, China

**Keywords:** MAFF, Mendelian randomization, Parkinson’s disease, protective factor, single-cell RNA sequencing

## Abstract

**Background:**

Parkinson’s disease (PD) is the second most prevalent neurodegenerative disorder. Although MAFF may be involved in PD, the mechanisms underlying this association remain unclear. This study aimed to investigate the potential mechanisms of MAFF in PD and identify novel therapeutic targets.

**Methods:**

PD-related datasets were obtained from public databases. The potential mechanisms of MAFF in PD were investigated using comprehensive gene expression profiling, receiver operating characteristic (ROC) analysis, Mendelian randomization (MR), functional enrichment analysis, immune infiltration analysis, drug prediction, molecular docking, and single-cell RNA sequencing (scRNA-seq). RT-qPCR and ELISA were then used to further validate MAFF expression in PD.

**Results:**

MAFF was significantly upregulated in PD samples across multiple datasets and in both mouse and clinical samples. ELISA showed significantly increased MAFF expression in serum samples from patients with PD. The area under the curve (AUC) values for MAFF exceeded 0.70 in both datasets, indicating good diagnostic potential. MR analysis suggested a potential causal association between MAFF and PD (OR = 0.9075, 95% CI = 0.8243–0.9990, *p* = 0.0477). Notably, MAFF was significantly enriched in the MAPK signaling pathway, suggesting its potential involvement in regulating key cellular processes, including inflammation, in PD. In addition, two differentially infiltrating immune cell types were identified, and MAFF was positively correlated with monocytes. Diphenylcyclopropenone showed the highest interaction score with MAFF, and molecular docking indicated binding between MAFF and diphenylcyclopropenone, with a binding energy of −5.5 kcal/mol. Finally, scRNA-seq analysis highlighted the central roles of pericytes and endothelial cells in PD. Pseudotime analysis showed that MAFF was highly expressed during the late differentiation stages of endothelial cells.

**Conclusion:**

Overall, this study demonstrated that MAFF may play an important role in PD. These findings provide insights into the molecular mechanisms of PD and may support the development of targeted therapeutic strategies.

## 1 Introduction

Parkinson’s disease (PD) is the second most common neurodegenerative disease, characterized by progressive death of dopaminergic neurons within substantia nigra and the formation of Lewy bodies (Tolosa et al., 2021). It is also the most rapidly growing neurological disorder, impacting the health of more than 10 million people around the world (Tinazzi et al., 2025). Being a progressive disease, PD worsens over time. The patients might suffer from various motor signs such as bradykinesia, tremor, and rigidity, as well as non-motor symptoms like depression, constipation, cognitive decline, and sleep problems (Tinazzi et al., 2025). Current treatments include pharmacotherapy, such as dopamine replacement therapy, and deep brain stimulation (DBS) surgery (Foltynie et al., 2024). However, these approaches only relieve the symptoms and have some disadvantages associated with limited efficacy of long-term pharmacotherapy, complications due to surgery, and the inability to treat non-motor symptoms of PD (Foltynie et al., 2024; Neumann et al., 2023). Therefore, the further study of the pathogenesis of PD and the development of new methods for treatment are necessary.

MAFF (MAF basic leucine zipper transcription factor F) is an important member of the small MAF basic leucine zipper (bZIP) family of transcription factors, which also includes MafG and MafK (Wang et al., 2020), and is mainly expressed in the nucleus. Even though MAFF does not contain any transcriptional activation domains, it can create heterodimerization with other transcription factors, for example, Nrf2 and exert transcriptional regulation (Li and Zhan, 2022). MAFF has been extensively studied in atherosclerosis (von Scheidt et al., 2021), cancers (Tsuchiya and Oura, 2018), chronic myeloid leukemia (Martinez-Hernandez et al., 2014), and diabetes (Shimohata et al., 2009). Regarding the neurodegenerative disorders, a number of papers have suggested connections between the expression of MAFF and the formation of Alzheimer’s disease (AD; Wang et al., 2020; Tian et al., 2022). In PD, a paper has suggested that MAFF may impair the function of Nrf2 by creating heterodimers with it and thus interfere with the expression of antioxidants leading to increased oxidative stress (Li and Zhan, 2022). Currently, the exact mechanism of MAFF action in PD is unknown, and more research on this topic is required.

MR uses genetic variants as instrumental variables (IVs) to infer causal relationships between exposures and disease outcomes (Birney, 2022; Dobrijevic et al., 2023). MR analyses have been widely used in the field of PD. For example, several studies used Mendelian randomization approach to evaluate the causality of the relationship between inflammation and PD (Bottigliengo et al., 2022). Therefore, it would be a powerful tool to test the causal relationship between MAFF and PD. scRNA-seq measures the expression of genes on a single-cell level, thus, allowing us to analyze cellular heterogeneity (Cheng et al., 2023). The role of scRNA-seq is not less important for the field of PD. Using scRNA-seq to profile gene expression in different cell types of the brain of PD patients could help reveal cellular heterogeneity in PD (Ma and Lim, 2021; Zhu et al., 2024).

By integrating transcriptomic, MR, and scRNA-seq data, this study investigated potential mechanisms associated with MAFF in PD and identified key cell types. In addition, RT-qPCR analyses of mouse and clinical samples were performed to further validate these findings. This study provides evidence for the role of MAFF in the pathological process of PD and offers a theoretical basis for exploring MAFF as a potential therapeutic target.

## 2 Materials and methods

### 2.1 Data collection

PD transcriptomic and scRNA-seq datasets were obtained from the Gene Expression Omnibus (GEO) database.^1^ Specifically, the GSE7621 and GSE20141 datasets, both based on the GPL570 platform, were included. GSE7621 contained 16 PD and 9 control nigral tissue samples, whereas GSE20141 contained 10 PD and 8 control samples. For the scRNA-seq analysis, GSE184950 (GPL24676) included 6 PD and 10 control nigral tissue samples.

For MR analysis, all data were retrieved from the IEU Open GWAS database.^2^ The dataset ebi-a-GCST90018894, which currently contains the largest number of single nucleotide polymorphisms (SNPs), included data from 480,018 European individuals, comprising 2,638 cases and 477,380 controls, with 24,194,622 SNPs. In addition, expression quantitative trait locus (eQTL) data related to the exposure factor, MAFF, were obtained from the IEU Open GWAS database for European populations.

### 2.2 Gene expression analysis and ROC analysis

MAFF expression was analyzed separately in the GSE7621 and GSE20141 datasets. The Wilcoxon test was used to compare MAFF expression between PD and control samples in each dataset, with *p* < 0.05 considered statistically significant.

To assess the diagnostic ability of MAFF in distinguishing PD from control samples, ROC curves were generated using the “pROC” package (v2.3.1; Zhong et al., 2024) for both the GSE7621 and GSE20141 datasets. The AUC was calculated, and an AUC value greater than 0.70 was considered to indicate acceptable diagnostic performance. To further improve the robustness of the results, the GSE7621 and GSE20141 datasets were merged. Batch effects were corrected using the ComBat function in the “sva” package (v3.46.0; Leek et al., 2012), and MAFF differential expression and ROC analysis were then evaluated in the combined dataset.

### 2.3 Data preprocessing for the MR study

In the MR analysis, MAFF eQTLs were defined as the exposure, and PD was defined as the outcome. Conventional MR analysis requires three core assumptions to be satisfied: (i) the independence assumption, which requires that IVs are not associated with any confounding factors; (ii) the relevance assumption, which requires that IVs are directly associated with the exposure; and (iii) the exclusion restriction assumption, which requires that IVs influence the outcome only through the exposure of interest.

IVs were first selected at *p* < 5 × 10^−6^ using the “TwoSampleMR” package (v0.6.1; Zhou et al., 2019). Linkage disequilibrium clumping was then performed (clump = TRUE, r^2^ = 0.001, kb = 10) to ensure SNP independence. Only SNPs with three or more associations were retained. Subsequently, only SNPs exclusively associated with the exposure were retained, whereas SNPs associated with the outcome were excluded. Effect alleles and effect sizes were then harmonized using the harmonise_data function. Finally, the strength of each SNP was assessed using F-statistics, with *F* > 10 indicating sufficient instrument strength. The F-statistic was calculated using the following equation:


$$F=\frac{R^{2}\times (n-2)}{1-R^{2}}$$

Here, R^2^ represents the proportion of exposure variance explained by the IVs, and n indicates the GWAS sample size for the exposure.

### 2.4 MR analysis, sensitivity analyses, and Steiger test

After IV screening, five algorithms were applied using the mr function to conduct the MR analysis: inverse-variance weighted (IVW; Burgess et al., 2013), MR-Egger (Bowden et al., 2015), weighted median (Hartwig et al., 2017), simple mode (Hemani et al., 2018), and weighted mode (Hartwig et al., 2017). Among these methods, the IVW method was considered the primary analysis, and the significance threshold for the MR analysis was set at P_IVW_ < 0.05. To visualize the results, several plots were generated using the “TwoSampleMR” package (v0.6.1). A scatter plot was used to assess the exposure-outcome association, a forest plot was used to visualize the effect estimates for the exposure, and a funnel plot was used to examine the symmetry of the causal-effect distribution.

To evaluate the robustness of the MR estimates, comprehensive sensitivity analyses were performed. Heterogeneity was assessed using the mr_heterogeneity function based on Cochran’s Q test (Lu et al., 2022). Horizontal pleiotropy was evaluated using the mr_pleiotropy_test function in “TwoSampleMR” (v0.6.1) and the mr_presso function in the “MRPRESSO” package (v1.0; Lou et al., 2023), with *p* > 0.05 indicating no significant pleiotropy. Subsequently, leave-one-out (LOO) analysis was conducted using the mr_leaveoneout function (Jin et al., 2022) to assess the stability of the MR estimates by iteratively excluding each IV.

Finally, the Steiger test was performed to verify the directionality of causal inference using the directionality_test function. A valid Steiger test result was defined as a correct causal direction (TRUE) and a *p*-value less than 0.05.

To improve the robustness of the MR findings, reverse MR analysis was performed with PD as the exposure and MAFF expression as the outcome, using the same analytical strategy as that used for the forward MR analysis.

### 2.5 Functional analyses

To explore potential interactions and functional relationships between MAFF and other genes, the GeneMANIA database^3^ was used to construct a gene–gene interaction (GGI) network. Gene set enrichment analysis (GSEA) was then conducted using the GSE7621 dataset to identify MAFF-associated pathways. After all genes were ranked according to their Spearman correlation with MAFF using the “psych” package (v2.2.9; Robles-Jimenez et al., 2021), GSEA was performed using “clusterProfiler” (v4.7.1.3; Yu et al., 2012) against the MSigDB “c2.cp.kegg.v7.4.symbols.gmt” gene set. Significant enrichment was defined as |NES| > 1 and *p* < 0.05, and the top five pathways ranked by *p*-value were visualized using “enrichplot” (v1.18.0; Xiong et al., 2022).

To identify pathway-level differences between PD and control samples in the GSE7621 dataset, Gene Set Variation Analysis (GSVA) was performed using the “GSVA” package (v1.42.0; Hanzelmann et al., 2013) with the MSigDB “c2.all.v7.5.1.symbols.gmt” gene set. Differential enrichment was then assessed using the “limma” package (v3.54.0; Ritchie et al., 2015), with pathways meeting |t| > 2 and *p* < 0.05 considered significantly altered.

### 2.6 Immune infiltration analysis

Using the ssGSEA algorithm implemented in the “GSVA” package (v1.42.0; Charoentong et al., 2017), the infiltration levels of 28 immune cell types were quantified in PD and control samples from the TCGA-LUAD dataset. Differential infiltration between groups was assessed using the Wilcoxon test, with *p* < 0.05 considered statistically significant. Significantly altered immune cell types were selected for downstream analyses. Correlation analysis was then performed to examine associations among these immune cell types and between immune cell types and MAFF, using thresholds of |cor| > 0.30 and *p* < 0.05.

### 2.7 Subcellular localization and tissue localization analyses

To explore the subcellular localization of MAFF, the GeneCards database^4^ was queried. In addition, MAFF expression across human tissues was analyzed using the Human Protein Atlas (HPA) database.^5^

### 2.8 Regulatory network analysis

To investigate potential regulatory mechanisms associated with MAFF in PD, regulatory network analyses were performed. Transcription factors targeting MAFF were predicted using the NetworkAnalyst database.^6^ The TF-mRNA regulatory network was constructed and visualized using Cytoscape (v3.10.2; Shannon et al., 2003).

In addition, microRNAs (miRNAs) targeting MAFF were predicted using the miRanda^7^ and miRDB^8^ databases. After key miRNAs were identified by intersecting the database predictions, their target lncRNAs were retrieved from the StarBase database^9^ using a clipExpNum > 4 cutoff. A comprehensive lncRNA-miRNA-mRNA network was then assembled and visualized using Cytoscape (v3.10.2).

### 2.9 Drug prediction and molecular docking

Potential PD drugs targeting MAFF were screened from the DSigDB database.^10^ The top 10 compounds ranked by interaction score were used to construct a MAFF-drug network in Cytoscape (v3.10.2).

To evaluate drug-MAFF binding affinity, the top-ranked drug was selected for molecular docking. The three-dimensional structure of the drug ligand was retrieved from PubChem,^11^ whereas the MAFF protein structure was obtained from the AlphaFold database.^12^ Molecular docking was performed using the CB-Dock website^13^ to calculate binding energy. A binding energy below −5 kcal/mol generally indicates strong drug-MAFF affinity, suggesting the potential for effective molecular interaction.

### 2.10 scRNA-seq analysis and functional enrichment analysis

The scRNA-seq dataset GSE184950 was analyzed using “Seurat” (v5.0.1; Ritchie et al., 2015). Quality control (QC) was performed to remove low-quality cells and genes. Cells with <200 genes and genes detected in <3 cells were excluded. Only cells meeting the following criteria were retained: nFeature_RNA < 3,000, nCount_RNA < 5,000, and percent.mt < 25%.

After QC, data were normalized using NormalizeData, followed by highly variable gene selection using FindVariableFeatures (method = “vst”) and labeling of the top 10 genes. Principal component analysis (PCA) was performed using the RunPCA function after the samples were normalized with the ScaleData function. The number of principal components retained was determined using the ElbowPlot and JackStraw functions (*p* < 0.05). Unsupervised clustering was performed using FindNeighbors/FindClusters (dims = 1:20, resolution = 0.5), followed by UMAP visualization and marker gene identification with FindAllMarkers (logfc.threshold = 0.5, min.pct = 0.25, return.thresh = 0.01). Cell types were annotated based on the CellMarker database,^14^ relevant literature (Fernandes et al., 2020), and the “SingleR” package (v2.4.1; Zheng et al., 2023). After the proportions of annotated cells in PD and control samples were visualized, MAFF expression was compared using the Wilcoxon test (*p* < 0.05). Cells with both significantly elevated MAFF expression (*p* < 0.05) and high expression levels were defined as key cells, suggesting their potential role in PD pathogenesis.

The biological functions of annotated cells were examined by functional enrichment analysis using “ReactomeGSA” (v1.12.0; Griss et al., 2020) in the GSE184950 dataset (*p* < 0.05). The top 10 pathways ranked by enrichment score difference were visualized.

### 2.11 Cell–cell communication and pseudotime analyses

To further investigate cellular crosstalk, the “CellChat” package (v1.6.1; Luo et al., 2023) was used to analyze cell–cell communication in the GSE184950 dataset, focusing on key cells and other annotated clusters. Cell–cell communication networks were established for both PD and control samples. Visualization focused on the number and strength of interactions between key cells and other annotated cell clusters. In addition, receptor-ligand pairs involved in these intercellular interactions were analyzed using the same “CellChat” package (v1.6.1).

To further characterize the key cells, secondary dimensionality reduction was performed using the same method described in Section 2.9. After key-cell subclusters were identified using FindAllMarkers (min.pct = 0.5, logfc.threshold = 0.25, test.use = “auc”), pseudotime analysis was performed using “Monocle” (v2.30.1; Qiu et al., 2017) to delineate their differentiation states and trajectories. This analysis also revealed dynamic MAFF expression patterns across pseudotime progression, providing insight into the developmental trajectories and differentiation states of these cells.

### 2.12 Sample collection from mice

Seven-week-old SPF mice, including 2 male and 3 female C57BL/6 mice in the control group and 2 male and 3 female B-hSNCA*A53T mice in the case group, were purchased from Bcgen (Beijing Biocytogen Co., Ltd.). After anesthesia, the following samples were collected: brain tissues fixed in 4% paraformaldehyde or snap-frozen in liquid nitrogen and stored at −80 °C; substantia nigra tissues snap-frozen and stored at −80 °C; and serum samples stored at −80 °C.

### 2.13 Reverse transcription quantitative PCR (RT-qPCR)

To validate MAFF expression, RT-qPCR was performed using substantia nigra tissue samples from 10 mice, including 5 controls and 5 cases. B-hSNCA*A53T mice showed significantly reduced latency to fall and total distance moved in the rotarod test, confirming impaired motor coordination (Supplementary Figure S3). After total RNA extraction with TRIzol reagent and quantification using a NanoPhotometer N50, cDNA was synthesized with the SureScript kit on an S1000TM Thermal Cycler. Using primers listed in Supplementary Table S1, qPCR was performed on a CFX Connect system with the following thermal profile: 95 °C for 1 min; 40 cycles of 95 °C for 20 s, 55 °C for 20 s, and 7 potential involvement 2 °C for 30 s.

In addition, 20 whole-blood samples, including 10 controls and 10 cases, were collected from the First Hospital of Shanxi Medical University for MAFF expression validation by RT-qPCR. All participants provided informed consent, and the study was approved by the institutional ethics committee (KYYJ-2023-120). The RT-qPCR results from clinical samples were consistent with those obtained from mouse samples. Primer sequences are listed in Supplementary Table S2. Relative mRNA expression was calculated using the 2^-ΔΔCT^ method. RT-qPCR data were then exported to Excel and imported into GraphPad Prism 10 (https://www.graphpad.com/) for statistical analysis and visualization.

### 2.14 Measurement of serum MAFF levels by ELISA

In this study, serum samples were collected from 4 case and 4 control blood samples from the clinic in the First Hospital of Shanxi Medical University, Taiyuan, China. All participants were given informed consent. The study had the approval of the First Hospital of Shanxi Medical University ethics committee (approval number: KYYJ-2023-120). Serum MAFF concentrations were quantified using a commercially available human MAFF ELISA kit (Enzyme Free, catalog No. MM-66129H2) according to the manufacturer’s instructions. Briefly, standards and samples were diluted appropriately before the assay. Then, 50 μL of horseradish peroxidase (HRP)-conjugated streptavidin was added to each well, followed by incubation for 60 min at 37 °C. After washing, the chromogenic reaction was initiated by adding 50 μL each of substrate A and substrate B, followed by incubation in the dark for 15 min. The reaction was terminated by adding 50 μL of stop solution, and the optical density (OD) was measured at 450 nm within 15 min using a microplate reader. MAFF concentrations were determined by interpolation from a standard curve generated using known standard concentrations.

### 2.15 Statistical analysis

Relative mRNA expression levels, calculated using the 2^-ΔΔCT^ method, were processed in Excel and subsequently used for statistical analysis and visualization in GraphPad Prism 10.^15^

## 3 Results

### 3.1 Expression pattern and diagnostic performance of MAFF

**Figure 1:**
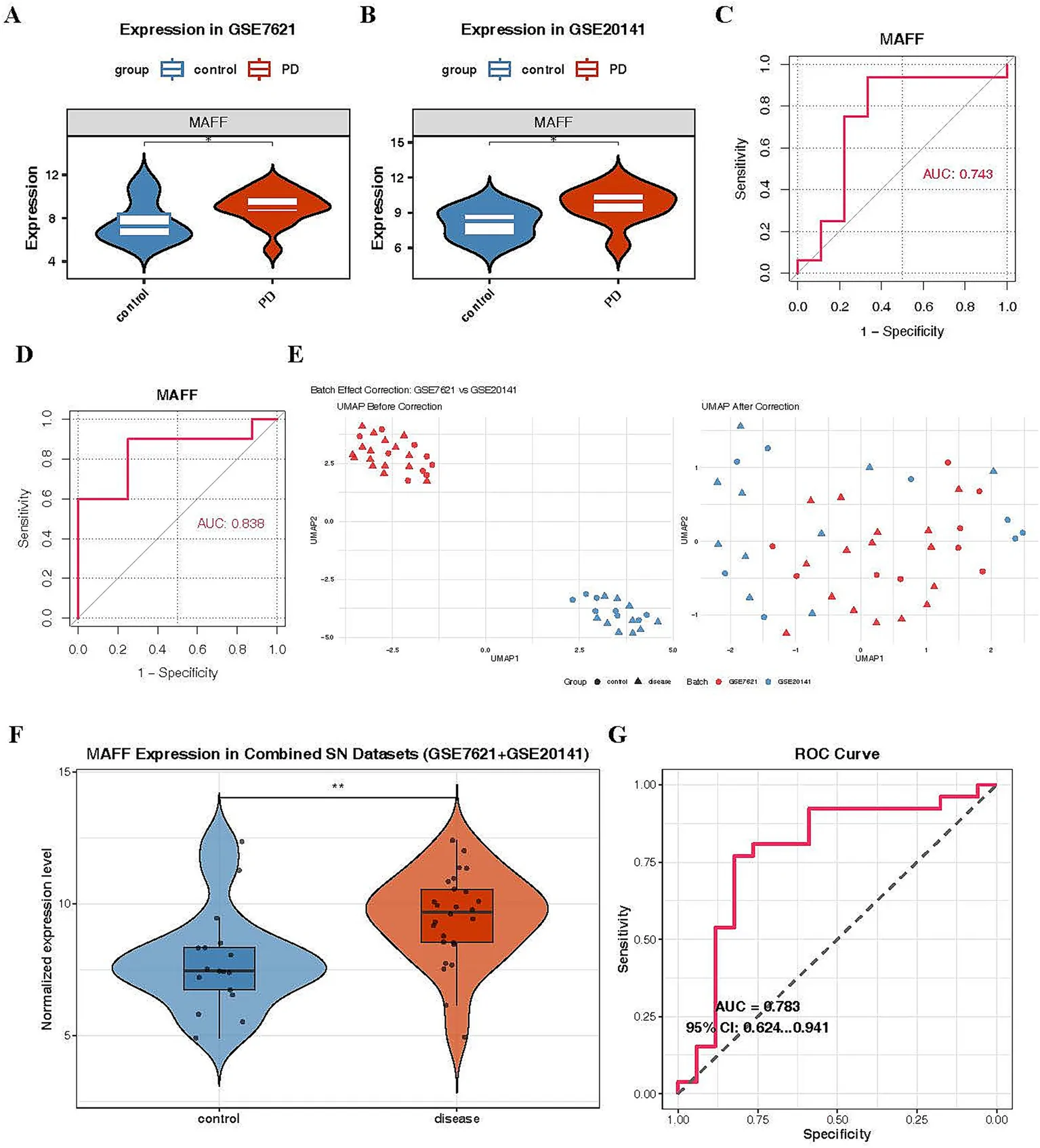
MAFF expression and diagnostic performance in PD datasets. **(A)** Gene expression in the GSE7621 dataset. **(B)** Gene expression in the GSE20141 dataset. **(C)** ROC analysis in the GSE7621 dataset. **(D)** ROC analysis in the GSE20141 dataset. **(E)** Batch effects between the GSE7621 and GSE20141 datasets were corrected using the ComBat function. **(F)** Gene expression in the combined dataset (GSE7621 + GSE20141). **(G)** ROC analysis in the combined dataset. **p < 0.01; *p < 0.05.

Gene expression analysis showed that MAFF was significantly upregulated in PD samples compared with control samples in both the GSE7621 and GSE20141 datasets (*p* < 0.05; Figures 1A,B). ROC analysis further demonstrated that MAFF had AUC values greater than 0.70 in both GSE7621 (AUC = 0.743) and GSE20141 (AUC = 0.838), indicating good diagnostic potential (Figures 1C,D). After the GSE7621 and GSE20141 datasets were merged and batch effects were corrected (Figure 1E), MAFF remained significantly upregulated in the disease group (Figure 1F) and showed good discriminatory ability between disease and control samples (AUC = 0.783; Figure 1G). These findings support the potential utility of MAFF as a diagnostic marker in PD research and clinical settings.

### 3.2 Potential causal relationship between MAFF and PD

**Figure 2:**
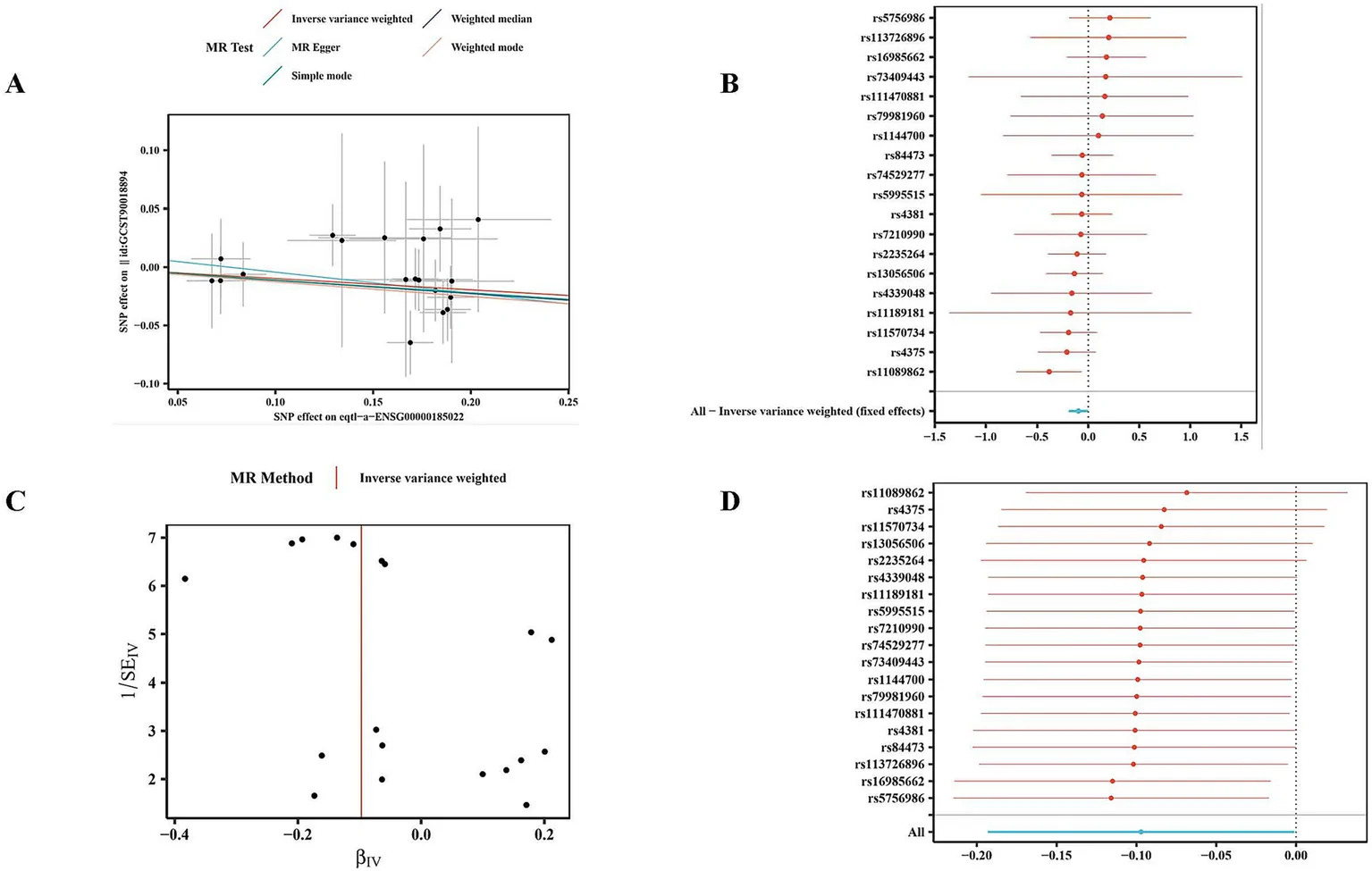
MR analysis of the causal effect of MAFF on PD risk. **(A)** Scatter plot for MAFF. **(B)** Forest plot analysis for MAFF. **(C)** Funnel plot for MAFF. **(D)** Results of LOO analysis.

A potential causal relationship between MAFF and PD was evaluated using MR analysis, sensitivity analyses, and Steiger testing. Specifically, the IVW method identified MAFF as a protective factor against PD (OR = 0.9075, 95% CI: 0.8243–0.9990, *p* = 0.0477; Table 1). This association was visually supported by the scatter plot, in which MAFF showed a negative slope (Figure 2A). Forest plot analysis also showed negative effect estimates for MAFF (MR effect sizes < 0; Figure 2B). Funnel plots further supported these findings by showing a symmetric distribution of IVs around the IVW line for MAFF, consistent with Mendel’s second law (Figure 2C).

**Table 1:** Results of the MR analysis for MAFF.

b	se	*p*	OR	OR_lci95	OR_uci95	SYMBOL	Method
−0.1820	0.1714	0.3030	0.8336	0.5958	1.1663	MAFF	MR Egger
−0.0971	0.0490	0.0477	0.9075	0.8243	0.9990	MAFF	Inverse variance weighted (IVW)
−0.1106	0.1024	0.2943	0.8953	0.7325	1.0943	MAFF	Simple mode
−0.1135	0.0700	0.1046	0.8927	0.7783	1.0239	MAFF	Weighted median
−0.1258	0.0714	0.0953	0.8818	0.7666	1.0144	MAFF	Weighted mode

Next, heterogeneity testing showed a *p*-value greater than 0.05 (*p* = 0.9267) using the fixed-effects IVW method, indicating no significant heterogeneity (Supplementary Table S3). The horizontal pleiotropy test showed no evidence of significant pleiotropy (*p* = 0.6116; Supplementary Table S3). LOO analysis confirmed the robustness of the MR results, with no significant deviations observed (Figure 2D). Finally, the Steiger test confirmed the correct causal direction for MAFF (correct causal direction = TRUE, *p* < 0.05), further supporting the reliability of the MR findings (Supplementary Table S4).

In addition, three SNPs (rs2736990, rs356182, and rs356220) were selected as IVs for the reverse MR analysis. No significant causal effect of PD on MAFF expression was detected (*β* = 0.0347, *p* = 0.323), with consistent nonsignificant estimates obtained using the MR-Egger and weighted median methods (all *p* > 0.05). The heterogeneity analysis showed absence of any heterogeneity among the SNPs (IVW Q = 0.666, Q_pval = 0.7169), and horizontal pleiotropy was not found to be present (MR-Egger intercept = 0.00293, SE = 0.0311, *p* = 0.940; Supplementary Figure S1A, Supplementary Table S5). Sensitivity analyses showed no variation among the pooled effect estimates after exclusion of each SNP one at a time using LOO method, and no individual SNP had a disproportionately large impact (Supplementary Figure S1B). The funnel plot indicated a nearly symmetrical distribution of the SNPs, suggesting absence of any publication bias (Supplementary Figure S1C). The Steiger test was positive in favor of the correct causal direction, but not significant (*p* = 0.130), providing very little support for precise causal direction (Supplementary Table S6). Taken together, all these results lend further weight to the strong Mendelian randomization findings showing the protective role of MAFF expression in relation to PD, suggesting that alterations in MAFF expression are not due to disease itself.

### 3.3 Functional characteristics and localization of MAFF

**Figure 3:**
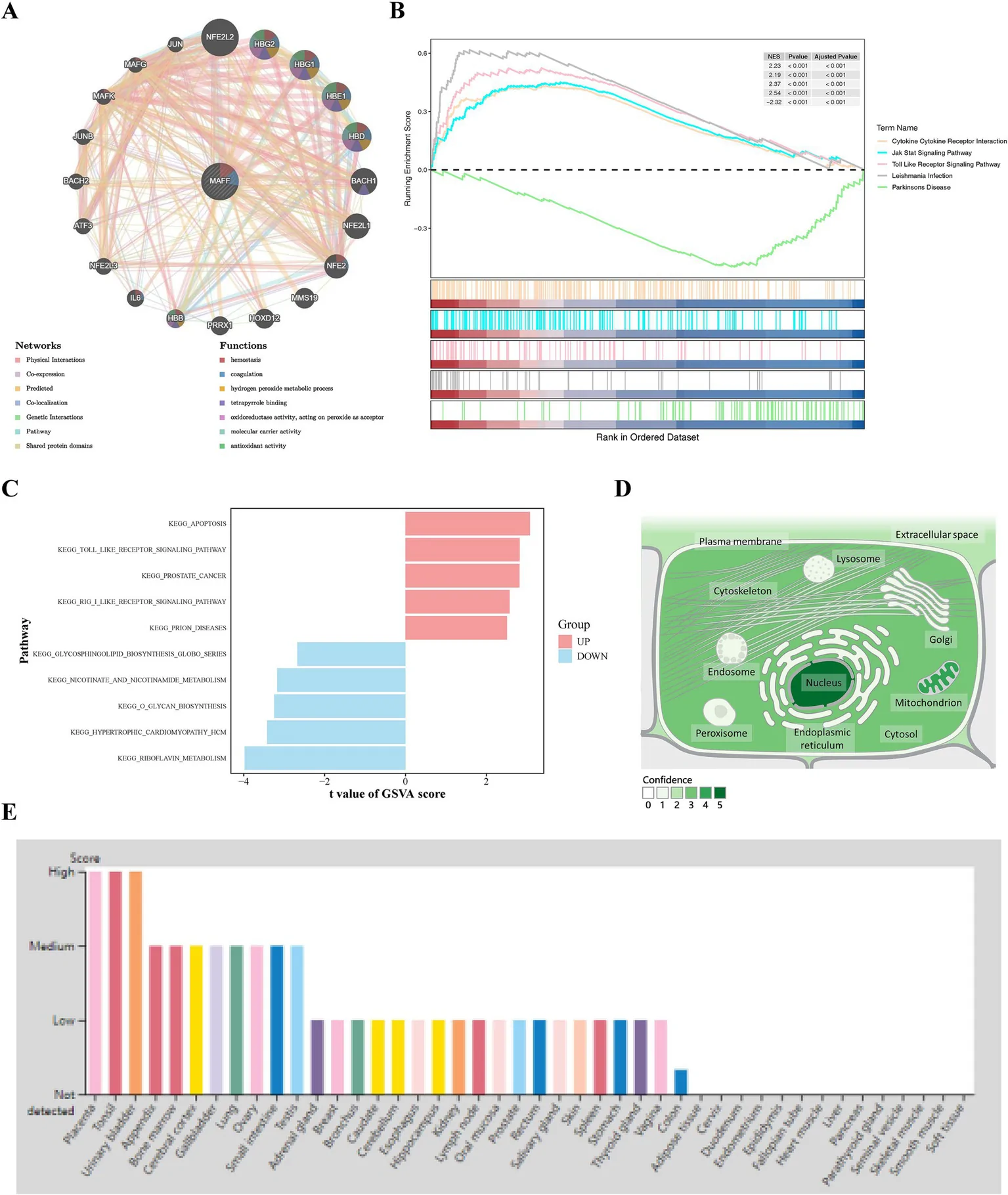
Functional annotation, pathway enrichment, and expression profiling of MAFF. **(A)** Gene interactions with MAFF identified using GeneMANIA. **(B)** Top five enriched pathways associated with MAFF. **(C)** Top five upregulated and top five downregulated pathways in PD. **(D)** Subcellular localization of MAFF. **(E)** Tissue distribution of MAFF.

Using GeneMANIA, 20 genes functionally associated with MAFF, including HBG2 and HBD, were identified. The gene interaction network was significantly enriched in key biological processes, including “homeostasis,” “coagulation,” and “hydrogen peroxide metabolic process,” suggesting a role in maintaining cellular and systemic balance (Figure 3A). GSEA showed that MAFF was significantly enriched in 64 pathways (|NES| > 1, *p* < 0.05), including the “MAPK_signaling_pathway” and “oxidative_phosphorylation” (Supplementary Table S7). The top five pathways were “cytokine-cytokine receptor interaction,” “JAK–STAT signaling pathway,” “Toll-like receptor signaling pathway,” “Leishmania infection,” and “PD” (Figure 3B). These findings suggest the potential involvement of MAFF in immune responses and neurodegenerative processes related to PD (Supplementary Table S7). Furthermore, GSVA showed significant differences between PD and control samples across 20 pathways (|t| > 2, *p* < 0.05; Supplementary Table S8). Specifically, the top five upregulated pathways, including “apoptosis” and “Toll-like receptor signaling pathway,” and the top five downregulated pathways, including “riboflavin metabolism” and “O-glycan biosynthesis,” were displayed, reflecting potential biological alterations in PD (Figure 3C).

Subcellular localization analysis showed that MAFF was predominantly localized to the nucleus (Figure 3D). In addition, MAFF was highly expressed in tissues such as the placenta, tonsil, and urinary bladder, suggesting its involvement in specific physiological functions (Figure 3E).

### 3.4 Immune infiltration differences between PD and control samples

**Figure 4:**
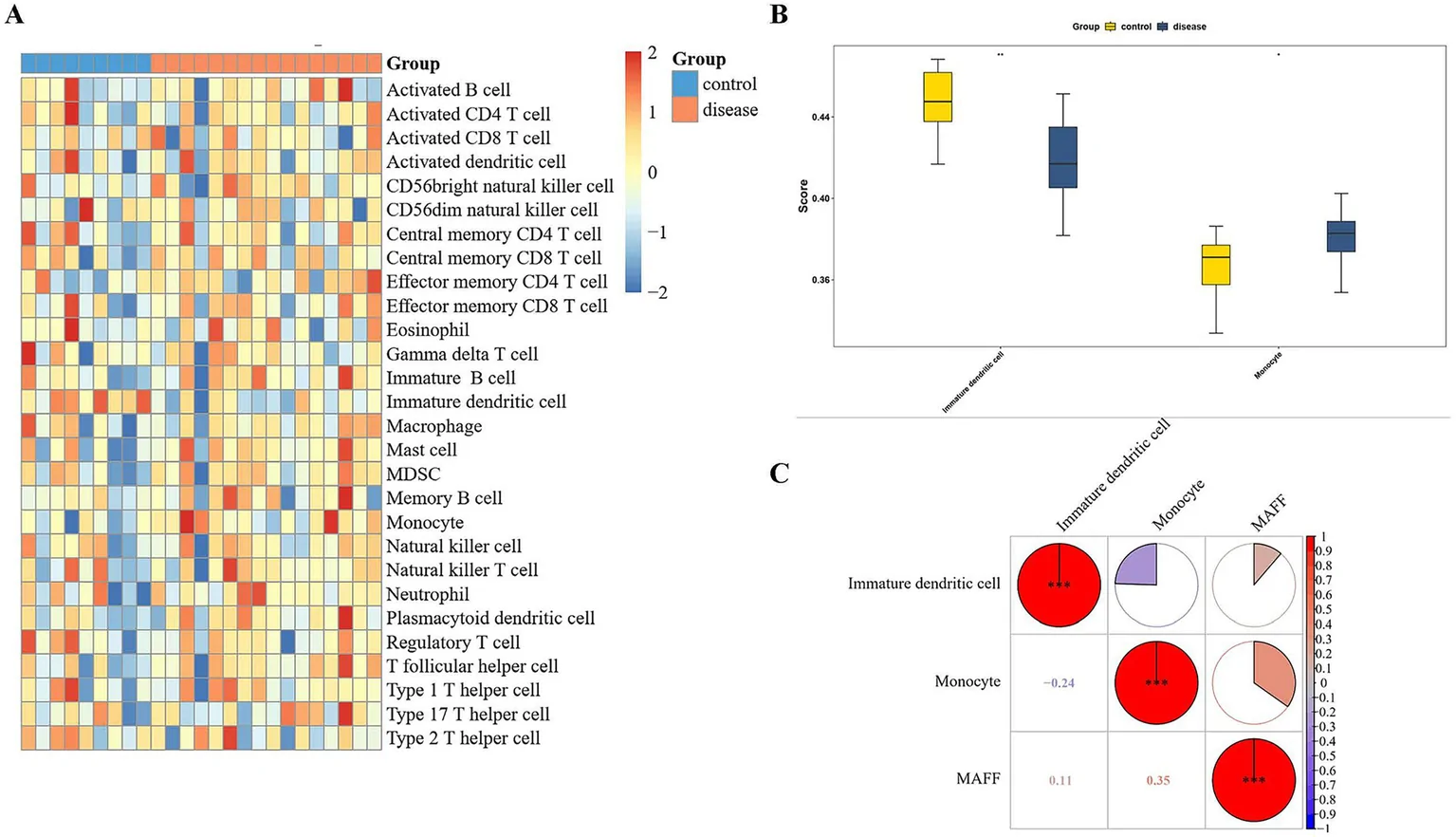
Immune infiltration landscape and correlation with MAFF in PD. **(A)** Immune infiltration scores in PD and control samples. **(B)** Two cell types showing significant differences between PD and control samples. **(C)** Correlation analysis among monocytes, immature dendritic cells, and MAFF. ns, *p* > 0.05; ***p < 0.001; **p < 0.01; *p < 0.05.

The immune infiltration landscape of 28 cell types was visualized using a stacked bar chart (Figure 4A). Comparative analysis identified two cell types with differential abundance between PD and control samples (*p* < 0.05; Figure 4B). Specifically, immature dendritic cells showed significantly lower infiltration in PD samples (*p* < 0.01), whereas monocytes showed significantly higher infiltration in PD samples (*p* < 0.05). Correlation analysis demonstrated a significant positive correlation between MAFF and monocytes (cor = 0.35, *p* < 0.001; Figure 4C).

### 3.5 Potential regulatory and therapeutic mechanisms in PD

**Figure 5:**
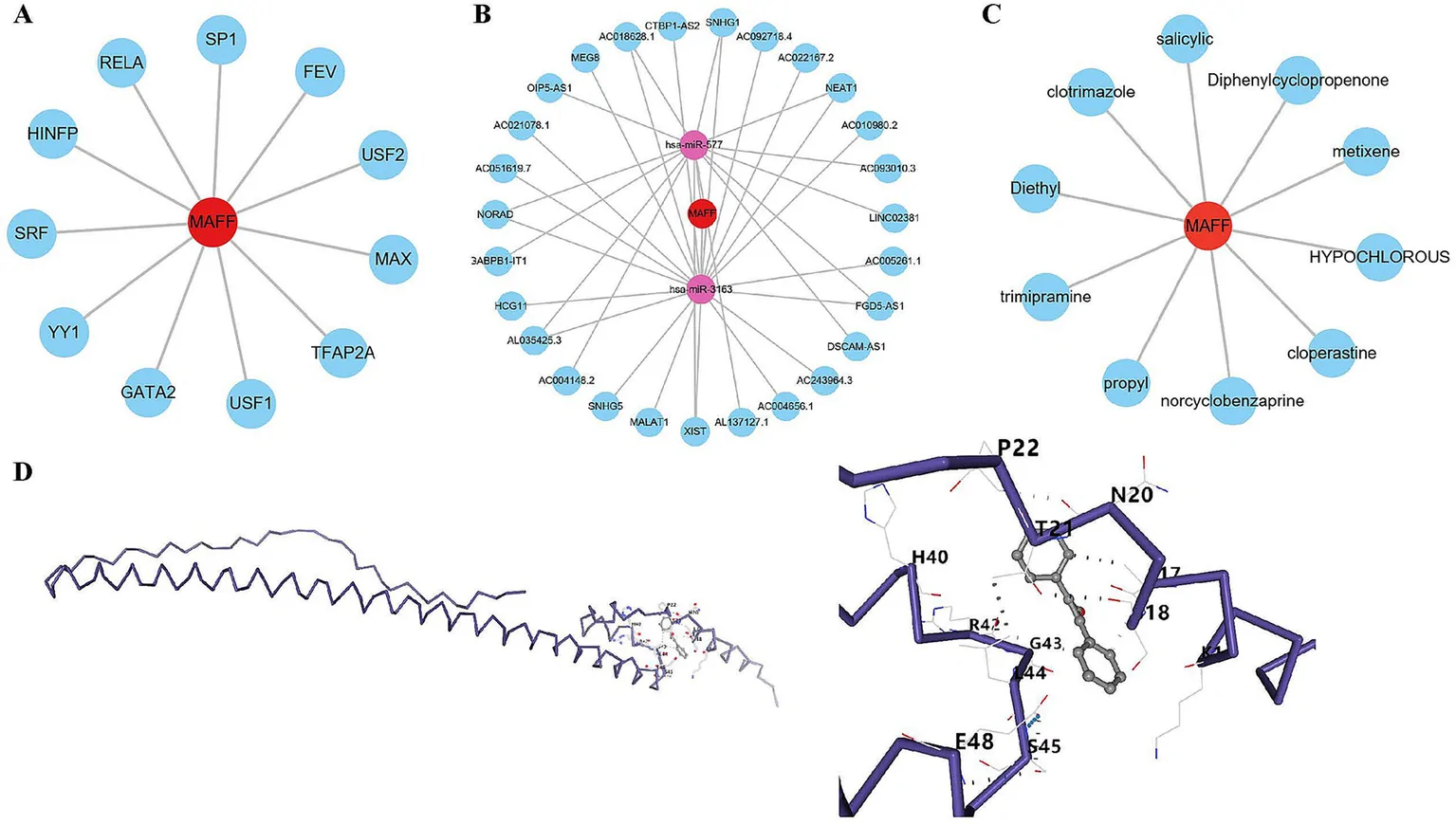
Regulatory network and drug prediction for MAFF. **(A)** Transcription factors targeting MAFF. **(B)** LncRNAs targeting key miRNAs that target MAFF. **(C)** Top potential therapeutic drugs targeting MAFF. **(D)** Molecular docking results between MAFF and diphenylcyclopropenone.

A comprehensive search identified 11 TFs targeting MAFF, including SP1, FEV, and USF2 (Figure 5A). In addition, two key miRNAs, hsa-miR-577 and hsa-miR-3163, were found to target MAFF. Further analysis identified 27 lncRNAs targeting these key miRNAs (clipExpNum > 4; Figure 5B). For example, NEAT1, SNHG1, XIST, and NORAD were found to co-target both key miRNAs. These regulatory networks provide insight into the molecular mechanisms of gene expression and highlight potential therapeutic targets, thereby improving understanding of gene regulation in PD.

In addition, potential therapeutic drugs targeting MAFF were comprehensively predicted, and 269 drugs were identified (Supplementary Table S9). The top 10 drugs were used to construct a MAFF-drug network, illustrating the relationships between MAFF and various drugs, such as MAFF-diphenylcyclopropenone and MAFF-metixene (Figure 5C). Diphenylcyclopropenone showed the strongest predicted interaction with MAFF. Molecular docking revealed a binding energy of −5.5 kcal/mol between MAFF and diphenylcyclopropenone, involving interactions with residues such as P22 and T21 (Figure 5D). Although these computational findings suggest that diphenylcyclopropenone may be a potential candidate, further experimental validation, including binding assays and cellular functional studies, is required to confirm its therapeutic relevance in PD.

### 3.6 Identification of pericytes and endothelial cells as key cell types

**Figure 6:**
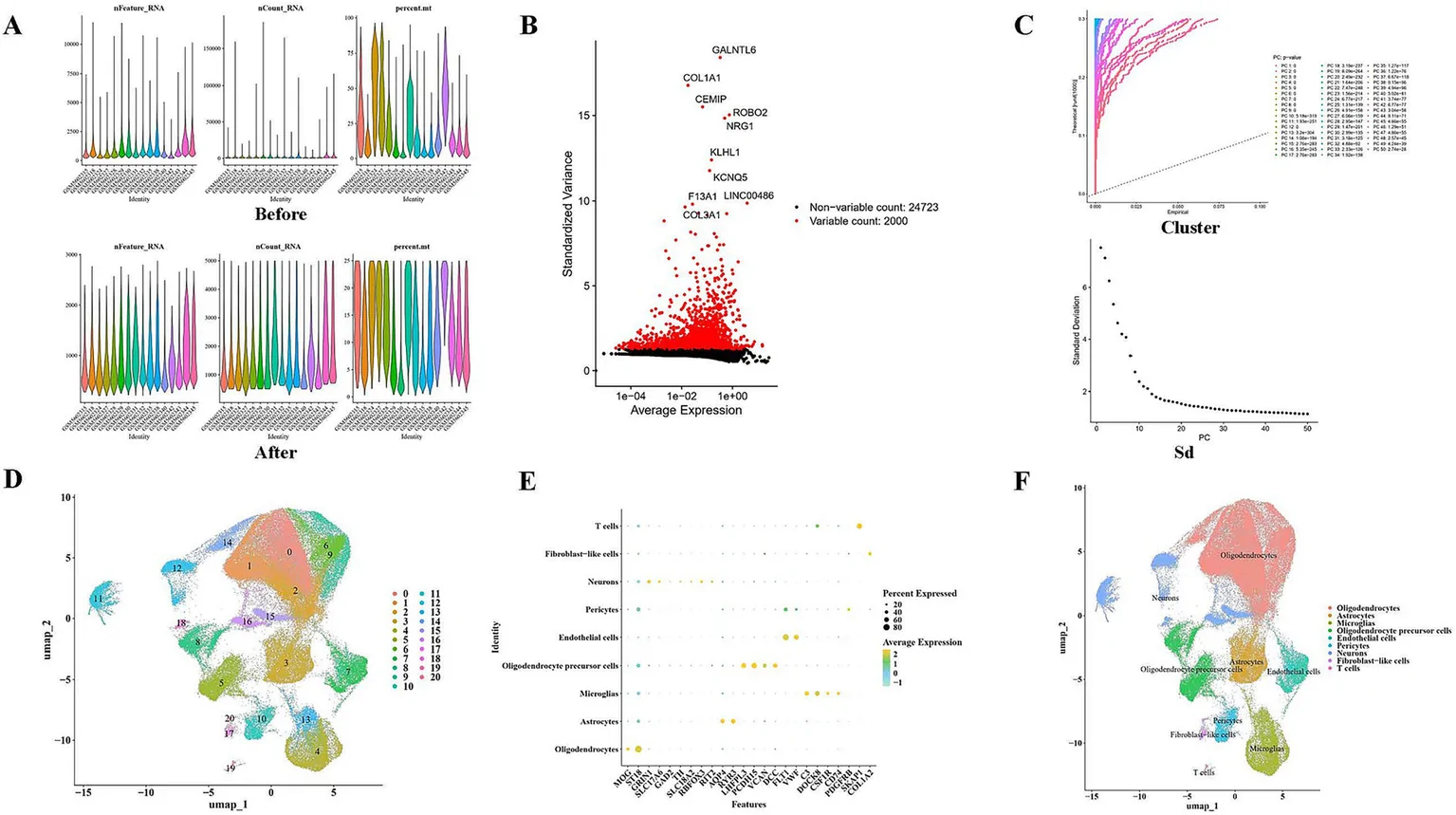
scRNA-seq profiling and cell type annotation. **(A)** Cells and genes before and after filtering. **(B)** Identified highly variable genes. **(C)** Top PCs selected for PCA. **(D)** Cell clusters. **(E,F)** Annotated cell types.

**Figure 7:**
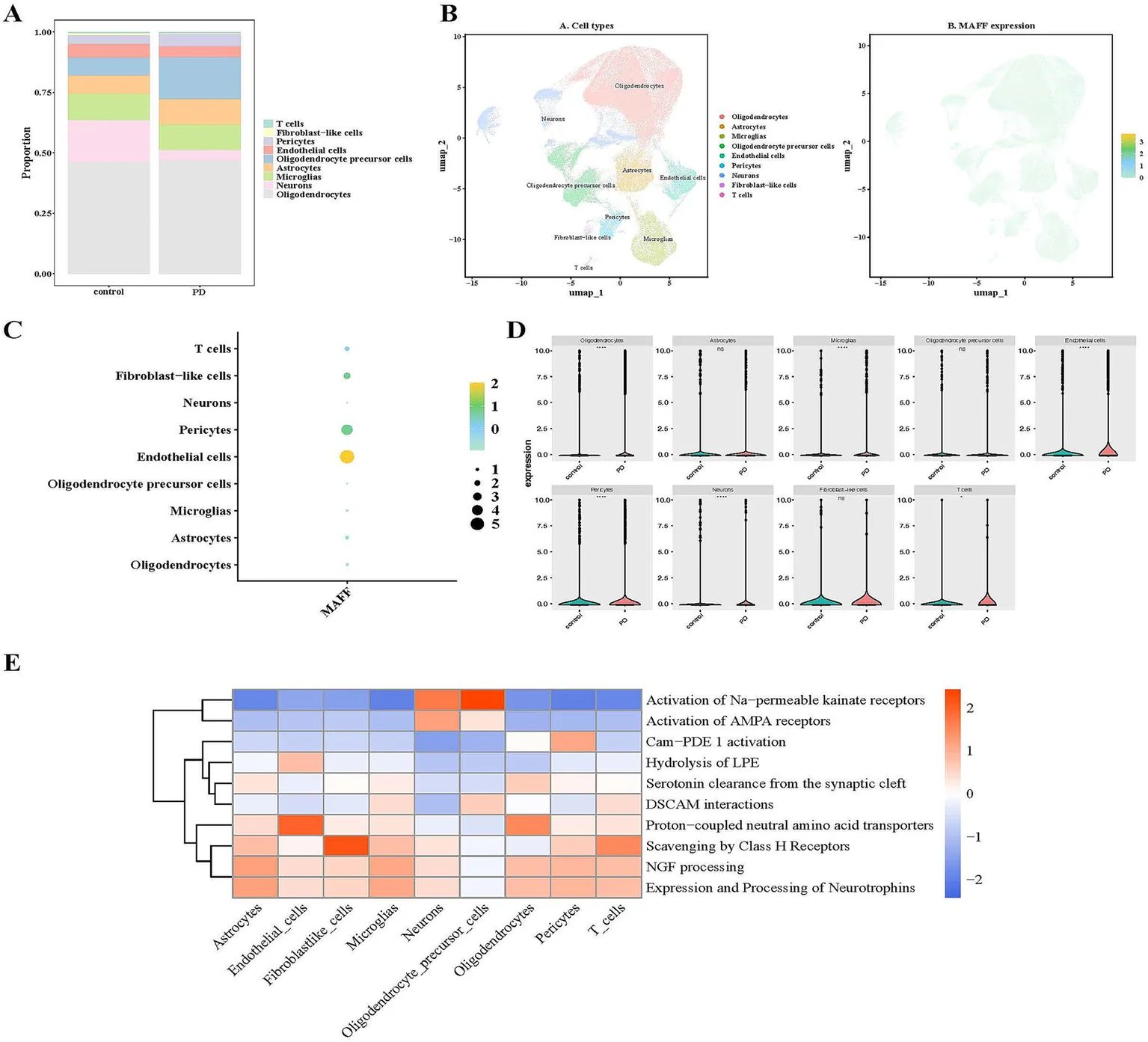
Single-cell landscape of MAFF expression and key cell type identification in PD. **(A)** Proportions of annotated cell types in PD and control samples. **(B)** MAFF expression across multiple cell types. **(C)** High MAFF expression in pericytes and endothelial cells. **(D)** Differential MAFF expression in annotated cells between PD and control samples. **(E)** Enriched pathways in annotated cells. ns, *p* > 0.05; ****p < 0.0001; *p < 0.05.

After exclusion of cells that were not eligible, a total of 116,105 cells and 26,723 genes were included (Figure 6A). Following the identification of 2,000 highly variable genes (HVGs; Figure 6B), PCA was performed, and the top 30 principal components were retained for subsequent analyses (*p* < 0.05; Figure 6C). UMAP visualization revealed 21 distinct cellular clusters (Figure 6D), which were subsequently annotated as various cell types, including oligodendrocytes, neurons, astrocytes, oligodendrocyte precursor cells, endothelial cells, microglia, pericytes, T cells, and fibroblast-like cells (Figures 6E,F). Among those annotated cell types, the largest number was the oligodendrocytes in both PD and control groups (Figure 7A). In addition, MAFF was widely expressed across multiple cell types (Figure 7B), and higher expression was observed in pericytes and endothelial cells (Figure 7C), with significant upregulation of MAFF in PD samples in these two cell types (Figure 7D). Overall, the present findings suggest that pericytes and endothelial cells are important cell types involved in PD pathology.

Function enrichment analysis determined the pathways that were enriched in each cell type. Endothelial cells were enriched for the proton-coupled neutral amino acid transporter pathway (*p* < 0.05), which suggests that these cells have a role in amino acid transport and metabolism. Pericytes had an enrichment in the Cam-PDE 1 activation pathway, which is related to signaling and vascular regulation (*p* < 0.05; Figure 7E). In summary, these findings provide insights into the mechanism of functions of these primary cell types in PD.

### 3.7 Interactions and differentiation trajectories of key cell types

**Figure 8:**
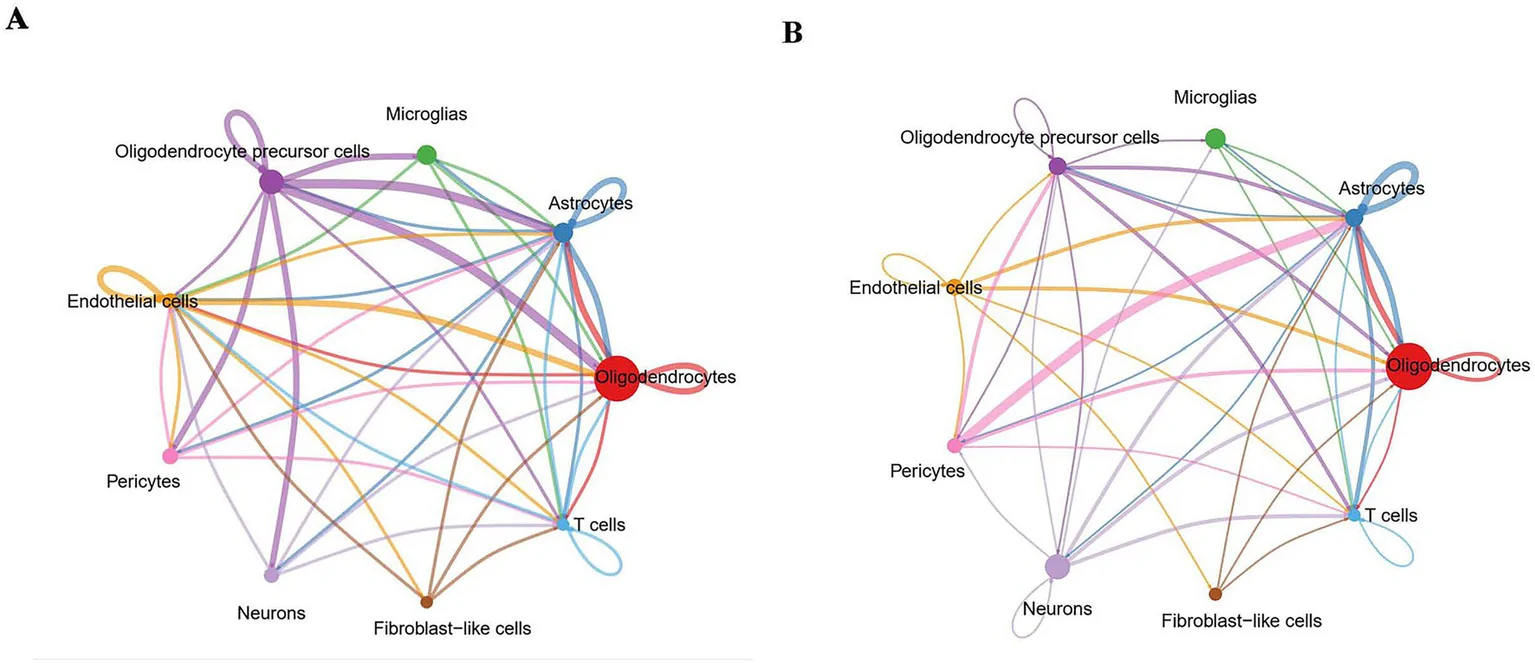
Cell communication analysis. **(A,B)** Cell–cell communication between key cells and other annotated cell types.

Cell–cell communication analysis revealed significant interaction networks between the key cells and other labeled cells. Notably, pericytes showed a greater level of communication with endothelial cells in PD samples as opposed to the controls. The communication of endothelial cells was observed with the pericytes, thus suggesting two-way communication between the two cell types in the PD samples (Figures 8A,B). Additionally, NRG3-ERBB4 signaling pathway was found to be the key mediator of the communication network for both PD samples and controls (Supplementary Figure S2A,B). These findings highlight the interactions between pericytes and endothelial cells and suggest their potential role in PD pathology.

**Figure 9:**
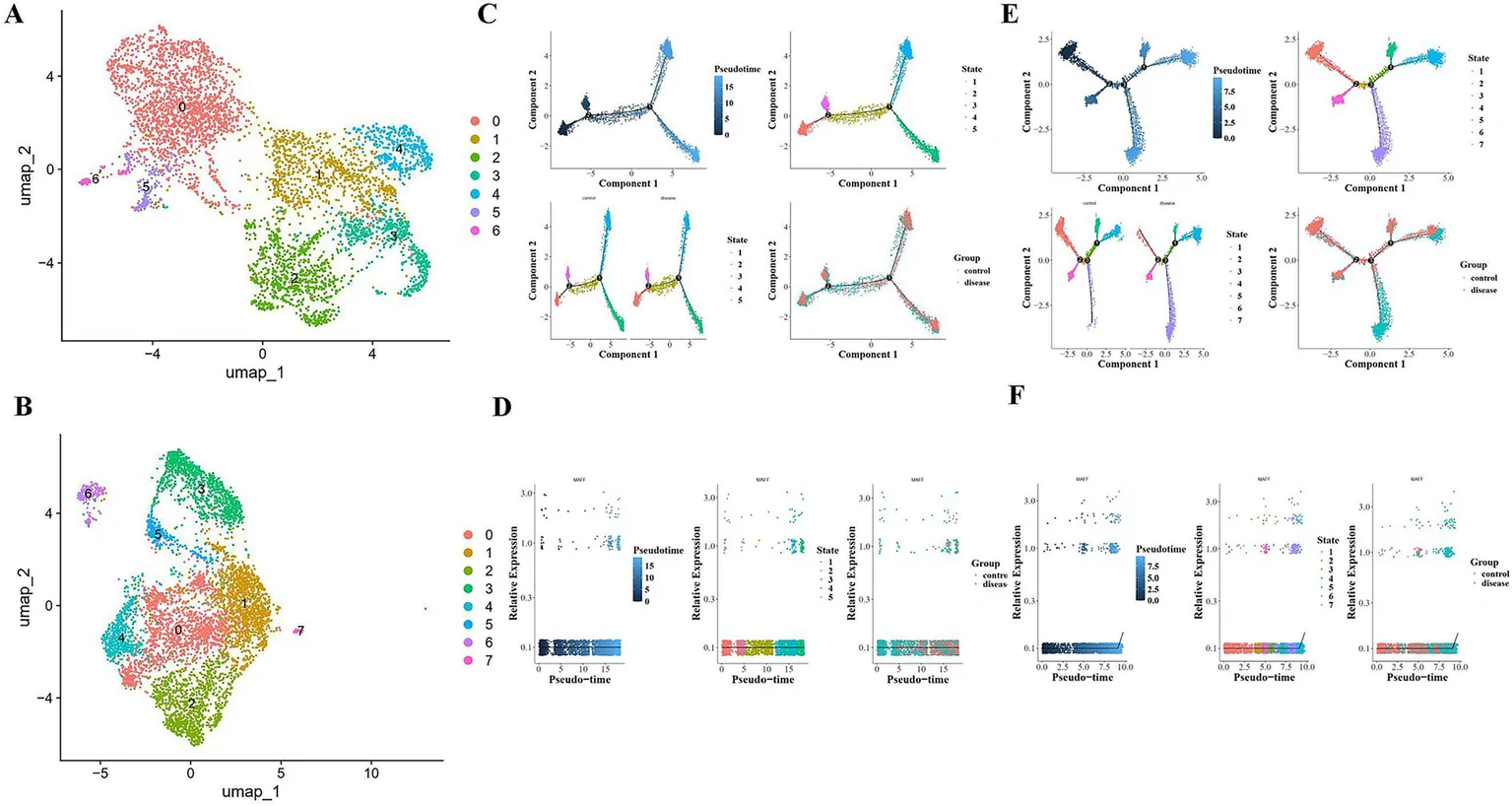
Subclustering and pseudotime analysis of pericytes and endothelial cells. **(A)** Pericyte subclusters. **(B)** Endothelial cell subclusters. **(C)** Differentiation trajectory of pericytes. **(D)** MAFF expression during pericyte differentiation. **(E)** Differentiation trajectory of endothelial cells. **(F)** MAFF expression during endothelial cell differentiation.

Additional dimensionality reduction analyses of the secondary type further classified pericytes into seven subclusters (subclusters 0–6; Figure 9A) and endothelial cells into eight subclusters (subclusters 0–7; Figure 9B). The results of such an analysis provided more information about the heterogeneity of these cell types with respect to PD. Pseudotime analysis showed the differentiation pathway of pericytes from left (dark blue) to right (light blue), consisting of five stages (Stages 1–5; Figure 9C). Stage 1 was considered to be the earliest stage of pericytes’ differentiation. The level of MAFF expression in the process of pericytes’ differentiation did not change significantly (Figure 9D), suggesting that MAFF may not be directly involved in the early phase of pericyte differentiation.

In contrast, pseudotime analysis of endothelial cells showed a differentiation trajectory from left (dark blue) to right (light blue), with cells classified into seven stages (Stages 1–7; Figure 9E). Stage 1 represented the earliest differentiation stage. During endothelial cell differentiation, MAFF expression increased markedly in the later stages, indicating its potential involvement in the later phases of endothelial cell maturation (Figure 9F). These findings implicate MAFF in the later stages of endothelial cell differentiation and suggest a potential role in PD pathology.

### 3.8 Experimental verification of MAFF expression

**Figure 10:**
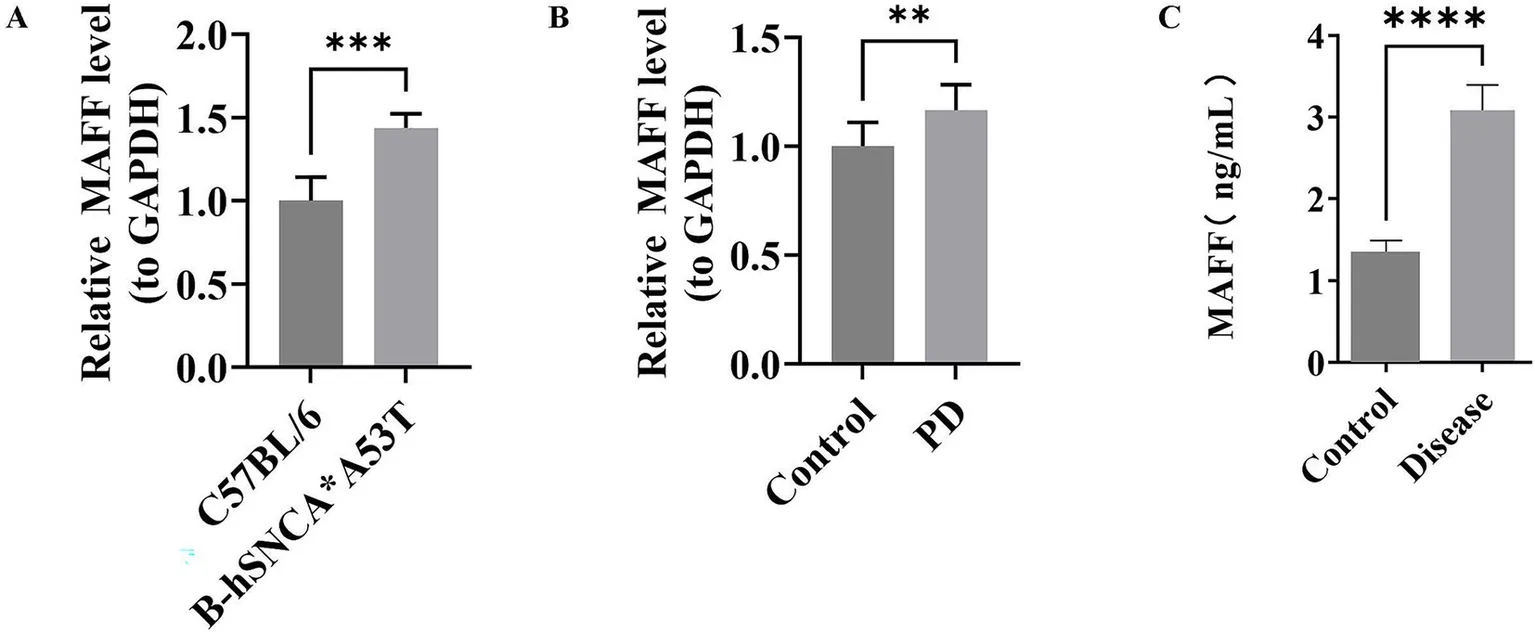
Experimental validation of MAFF expression in PD models and clinical samples. **(A)** RT-qPCR analysis of mouse samples. **(B)** RT-qPCR analysis of clinical samples. **(C)** ELISA analysis of clinical samples. ****p < 0.0001; ***p < 0.001; **p < 0.01.

RT-qPCR analysis of mouse samples confirmed that MAFF was significantly upregulated in B-hSNCA*A53T transgenic mice compared with C57BL/6 controls (Figure 10A). Similarly, RT-qPCR analysis of clinical whole-blood samples validated the significant upregulation of MAFF in patients with PD compared with controls (Figure 10B). ELISA revealed significantly elevated MAFF expression in serum samples from case group compared with control groups (Figure 10C).

## 4 Discussion

This study provides new insight into potential therapeutic targets for PD through multi-omics analysis and highlights the potential value of increasing MAFF levels to reduce PD risk. In addition, given the lack of specific diagnostic markers for PD, our findings suggest that MAFF may serve as a potential biomarker. Although differential expression analysis showed that MAFF was upregulated in PD, MR analysis suggested a protective role of higher MAFF expression, implying that this upregulation may represent a compensatory protective response rather than a pathogenic driver. Reverse MR analysis further showed no causal effect of PD on MAFF expression, supporting the robustness of the forward MR findings and reinforcing MAFF as a potential therapeutic target.

MAFF, a member of the basic region leucine zipper (bZIP) family of transcription factors, functions as either a transcriptional repressor or activator. Garcia et al. reported concurrent downregulation and hypermethylation of MAFF in osteoarthritic cartilage, indicating its role as a regulator of inflammation in osteoarthritis (Alvarez-Garcia et al., 2016). MAFF has also been reported to participate in cancer development by regulating antioxidant responses (Motohashi and Yamamoto, 2007). Another study identified MAFF as a hub regulator in a liver network relevant to atherosclerosis and coronary artery disease (CAD), affecting CAD risk by inducing specific LDLR expression (von Scheidt et al., 2021). No studies have directly defined the role of MAFF in PD, and only one study has suggested that MAFF may participate in the pathophysiology of PD by forming heterodimers with Nrf2 and exacerbating oxidative stress-related damage (Wang et al., 2017). In the present study, MR analysis identified a significant causal relationship between MAFF and PD, with MAFF acting as a protective factor against PD. High MAFF expression in patients with PD may reflect a cellular stress response to these pathological processes (Wang et al., 2020; Zhai et al., 2025), potentially helping to alleviate inflammatory injury and maintain cellular homeostasis through the upregulation of antioxidant gene expression. However, this protective mechanism may be insufficient to fully counteract PD pathology, allowing disease progression to continue. Moreover, MAFF may have potential as a biomarker for PD. First, MAFF was highly expressed in PD samples, and ROC curve analysis showed good diagnostic performance, with AUC values greater than 0.70 in both datasets, suggesting potential utility in early diagnosis and clinical intervention. Second, the observed protective effect identifies MAFF as a potential therapeutic target. Regulation of MAFF expression or its downstream pathways may therefore offer a basis for developing therapeutic strategies to delay PD progression.

The functional enrichment analysis also showed that the MAFF protein was highly enriched in the cytokine-cytokine receptor interaction and the associated signaling pathways, thus adding another aspect to the possibility of involvement of the protein in the pathophysiology of PD. In terms of individual cells, the pericytes and endothelial cells were found to be the primary cell types involved in the process of PD pathophysiology. MAPK signaling pathway forms one of the major signal transduction pathways in the cells that link the extracellular signals to the nuclear genes (Lin et al., 2025; Xue et al., 2022). Research has shown that the MAPK signaling pathway is vital in several neurological diseases. For instance, during traumatic brain injury, suppression of the MAPK signaling pathway reduces both apoptosis and inflammation, leading to neuroprotection (Li et al., 2022; Liu et al., 2022). In AD, p38 MAPK can promote Aβ-induced neuroinflammatory responses and neuronal apoptosis (Hosseini et al., 2024; Thakur et al., 2023). The MAPK pathway is also closely associated with PD. A recent study showed that the MEK1/2–ERK2 pathway regulates α-synuclein levels through a dual mechanism and that MEK1/2 inhibitors reduce intracellular α-synuclein levels, improving neuronal death and behavioral phenotypes in a mouse model of PD (Wang et al., 2025). As a transcription factor, MAFF binds to DNA through a basic leucine zipper structure and participates in the formation of the AP1 complex (Gazon et al., 2017). ERK and AP1 are key molecules in cellular signaling pathways and regulate a wide range of biological functions. For example, ERK enhances AP1 transcriptional activity by phosphorylating the JUN protein (Kushner et al., 2000). Conversely, AP1 can affect the ERK pathway by regulating the expression of specific genes (Kim et al., 2018; Eriksson et al., 2005). Therefore, we speculate that MAFF may regulate α-synuclein levels through the AP1/ERK signaling pathway, thereby participating in the pathophysiological process of PD. Further experimental studies are required to confirm this hypothesis.

Endothelial cells serve as vital constituents of the blood–brain barrier (BBB), helping maintain the integrity of the BBB through their interaction with pericytes and astrocytes (Percario et al., 2020). In the context of PD, it was shown by Dieriks et al. (Robea et al., 2020) and Stevenson (Zierfuss et al., 2024) that pericytes have the ability to internalize aggregated fibrils of α-synuclein. Dysfunction of endothelial cells may lead to the impairment of BBB integrity, making it easier for inflammatory molecules to enter the brain (Dieriks et al., 2022). Our study further supports the involvement of endothelial cells and pericytes in BBB dysfunction in PD. MAFF was highly expressed at the terminal stage of endothelial cell differentiation, suggesting an important regulatory role during late endothelial maturation. MAFF may contribute to BBB integrity by regulating specific gene expression programs and promoting endothelial cell maturation.

Monocytes, a major type of white blood cell in peripheral blood, have several important immune functions, including phagocytosis of pathogens and cellular debris, antigen presentation for adaptive immune activation, participation in inflammatory responses, and promotion of tissue repair (Stevenson et al., 2022; Cohen et al., 2024). In PD, monocytes are thought to exacerbate neurodegenerative disease progression by secreting proinflammatory cytokines (Coillard and Segura, 2021). In this study, MAFF showed a significant positive correlation with monocytes (cor = 0.35, *p* < 0.001). Given the established role of monocytes in regulating inflammatory processes, MAFF expression in these cells suggests a potential mechanism through which MAFF may influence inflammation.

Diphenylcyclopropenone (DPCP) is an immunomodulating topical agent used mainly to treat autoimmune and skin diseases. DPCP induces allergic contact dermatitis and may also affect immune systems in various ways. Therapeutic activity has been observed in different disease models (Nowicka et al., 2018; Abd El-Magid et al., 2023; Jativa et al., 2024; Grozdanov et al., 2014). The drug prediction study showed that DPCP had the highest score of interaction with MAFF. Molecular docking studies showed a good binding energy between MAFF and DPCP of −5.5 kcal/mol. This result suggests that there may be some translational significance of MAFF. However, further *in vitro* binding assays and cellular functional validation are required.

This study highlights a considerable increase in the MAFF expression in patients with PD, which was confirmed through RT-qPCR and ELISA. Also, the potential diagnostic significance of MAFF was confirmed using ROC curve analysis. MR analysis points out that there is a protective function of MAFF on PD. In addition, some insights into the mechanisms of regulation of the results obtained were provided in this work using enrichment and other omics methods. Two important cell types were determined at the cellular level – pericytes and endothelial cells. They can be involved in the development of PD. Nevertheless, despite some important findings, certain limitations should be considered. First of all, the small number of samples used in this study may affect the statistical significance of the results. Second, transcriptomic data may be subject to methodological bias. Third, further validation in other key brain regions (e.g., striatum and putamen) and additional independent datasets is warranted. Fourth, although the upregulation of MAFF in PD was validated at both the mRNA and protein levels, direct functional interventions, such as *in vitro* or *in vivo* knockdown or overexpression models, were not performed in the current study because of experimental constraints. Therefore, the specific mechanisms by which MAFF regulates oxidative stress, inflammation, and α-synuclein pathology in PD remain largely predictive. Further functional studies *in vivo* and *in vitro* are needed to fully elucidate its biological mechanisms and improve clinical application.

In summary, integrated multi-omics analysis and RT-qPCR validation in mouse and clinical samples support MAFF as a promising biomarker. Future research should further clarify the characteristics of MAFF expression at different stages of PD and the MAPK-related downstream signaling pathways to support the development of individualized therapies for patients with PD.

## Data Availability

The original contributions presented in the study are included in the article/Supplementary material, further inquiries can be directed to the corresponding author.

## Footnotes

1
https://www.ncbi.nlm.nih.gov/geo/

2
https://gwas.mrcieu.ac.uk/

3
http://genemania.org/

4
https://www.genecards.org/

5
https://www.proteinatlas.org/

6
https://www.networkanalyst.ca/

7
http://mirtoolsgallery.tech/mirtoolsgallery/node/1055

8
https://mirdb.org/

9
https://starbase.sysu.edu.cn/

10
https://dsigdb.tanlab.org/

11
https://pubchem.ncbi.nlm.nih.gov/

12
https://alphafold.ebi.ac.uk/entry/

13
https://cadd.labshare.cn/cb-dock/php/blinddock.php

14
http://xteam.xbio.top/CellMarker/

15
https://www.graphpad.com/
