# Supplementary material for: MAFF as a potential novel protective factor against Parkinson’s disease: evidence from Mendelian randomization and single-cell RNA sequencing
Source: Front Aging Neurosci. 2026 Sep 11;18:1759121. doi: 10.3389/fnagi.2026.1759121 (PMC13612465; doi:10.3389/fnagi.2026.1759121)
Supplement: Supplementary file 1 [file Data_Sheet_1.PDF]

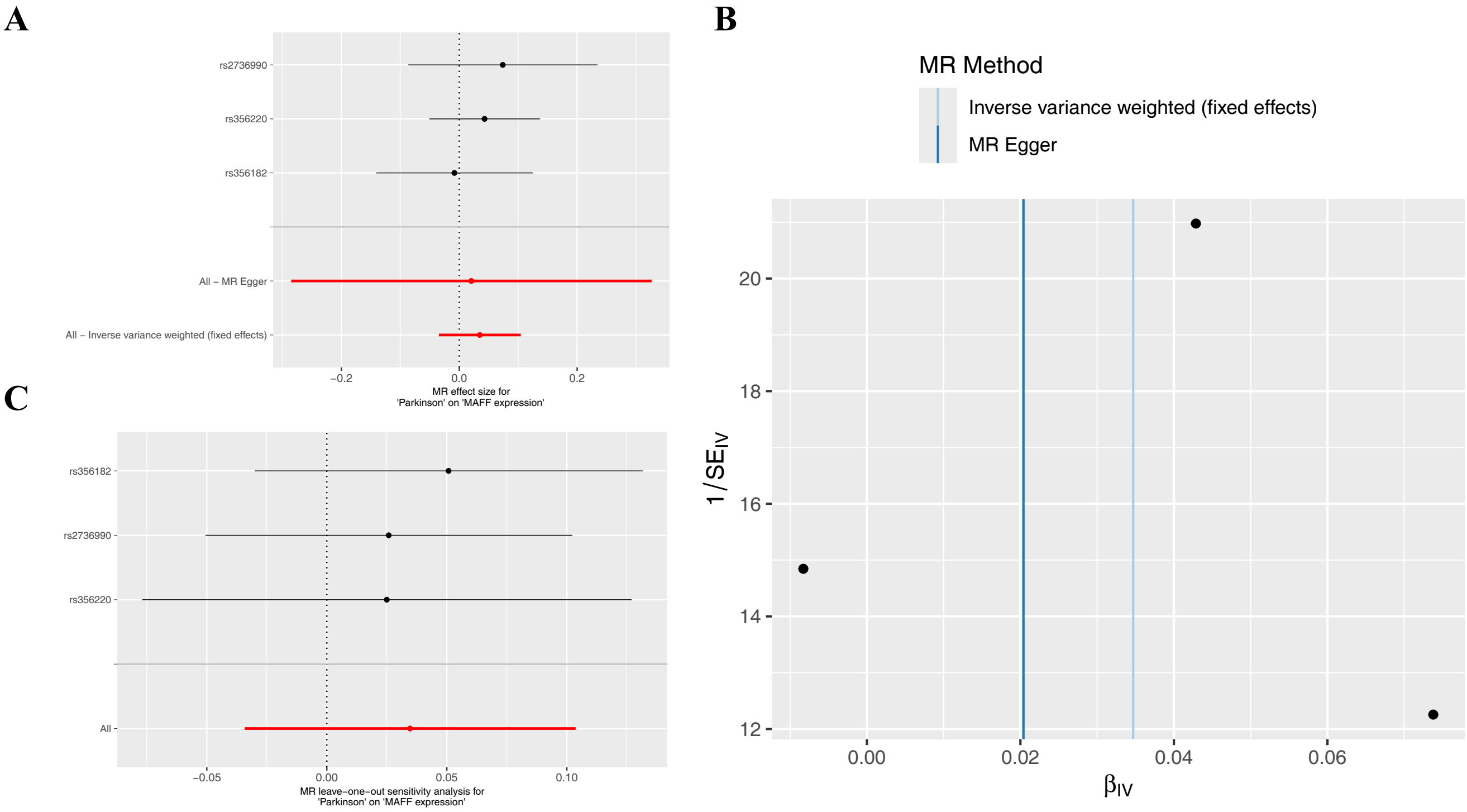
A
rs2736990
rs356220
rs356182
All – MR Egger
All – Inverse variance weighted (fixed effects)
−0.2
0.0
0.2
MR effect size for
'Parkinson' on 'MAFF expression'
B
MR Method
Inverse variance weighted (fixed effects)
MR Egger
$1/SE_{IV}$
12
14
16
18
20
0.00
0.02
0.04
0.06
β_{IV}
C
rs356182
rs2736990
rs356220
All
−0.05
0.00
0.05
0.10
MR leave–one–out sensitivity analysis for
'Parkinson' on 'MAFF expression'
