# Supplementary material for: MAFF as a potential novel protective factor against Parkinson’s disease: evidence from Mendelian randomization and single-cell RNA sequencing
Source: Front Aging Neurosci. 2026 Sep 11;18:1759121. doi: 10.3389/fnagi.2026.1759121 (PMC13612465; doi:10.3389/fnagi.2026.1759121)
Supplement: Supplementary file 2 [file Data_Sheet_2.PDF]

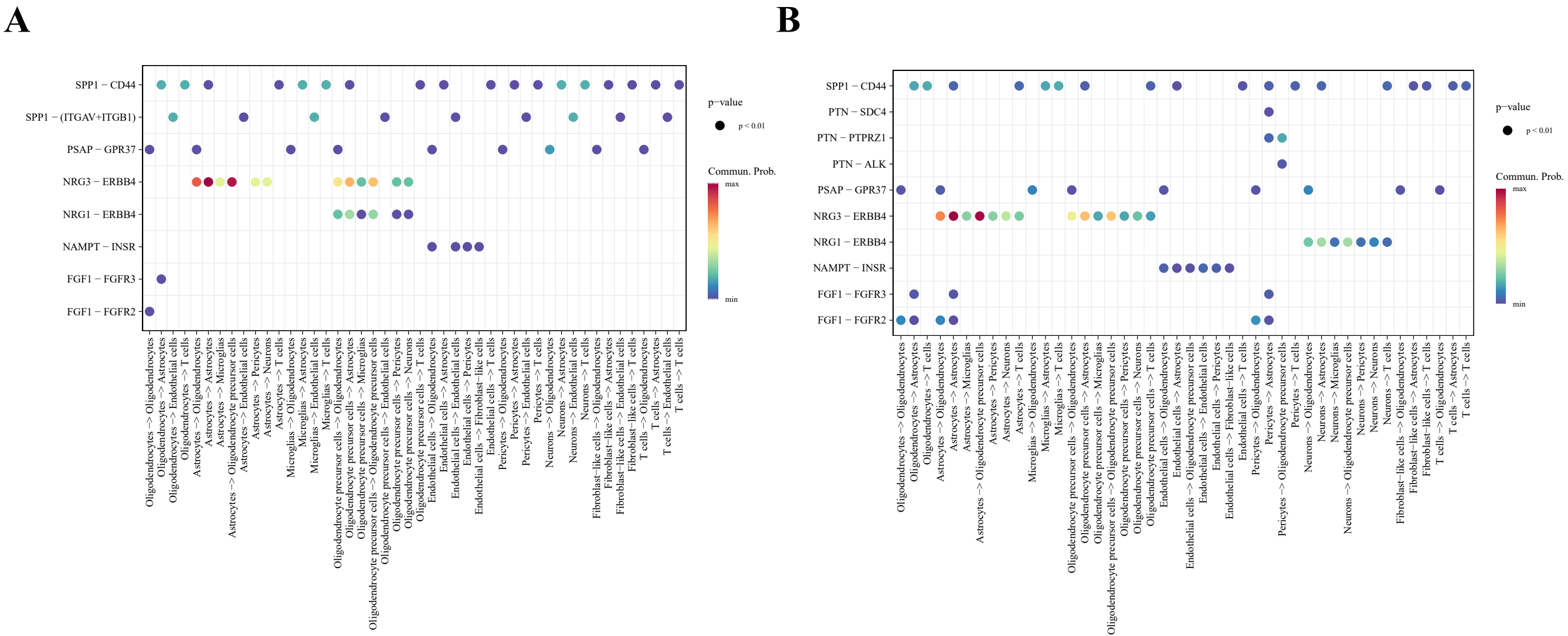

A
SPP1 − CD44
SPP1 − (ITGAV+ITGB1)
PSAP − GPR37
NRG3 − ERBB4
NRG1 − ERBB4
NAMPT − INSR
FGF1 − FGFR3
FGF1 − FGFR2
Oligodendrocytes −> Oligodendrocytes
Oligodendrocytes −> Astrocytes
Oligodendrocytes −> Endothelial cells
Oligodendrocytes −> T cells
Astrocytes −> Oligodendrocytes
Astrocytes −> Astrocytes
Astrocytes −> Microglias
Astrocytes −> Oligodendrocyte precursor cells
Astrocytes −> Endothelial cells
Astrocytes −> Pericytes
Astrocytes −> Neurons
Astrocytes −> T cells
Microglias −> Oligodendrocytes
Microglias −> Astrocytes
Microglias −> Endothelial cells
Microglias −> T cells
Oligodendrocyte precursor cells −> Oligodendrocytes
Oligodendrocyte precursor cells −> Astrocytes
Oligodendrocyte precursor cells −> Microglias
Oligodendrocyte precursor cells −> Oligodendrocyte precursor cells
Oligodendrocyte precursor cells −> Endothelial cells
Oligodendrocyte precursor cells −> Pericytes
Oligodendrocyte precursor cells −> Neurons
Oligodendrocyte precursor cells −> T cells
Endothelial cells −> Oligodendrocytes
Endothelial cells −> Astrocytes
Endothelial cells −> Endothelial cells
Endothelial cells −> Pericytes
Endothelial cells −> Fibroblast−like cells
Endothelial cells −> T cells
Pericytes −> Oligodendrocytes
Pericytes −> Astrocytes
Pericytes −> Endothelial cells
Pericytes −> T cells
Neurons −> Oligodendrocytes
Neurons −> Astrocytes
Neurons −> Endothelial cells
Neurons −> T cells
Fibroblast−like cells −> Oligodendrocytes
Fibroblast−like cells −> Astrocytes
Fibroblast−like cells −> Endothelial cells
Fibroblast−like cells −> T cells
T cells −> Oligodendrocytes
T cells −> Astrocytes
T cells −> Endothelial cells
T cells −> T cells
p−value
p < 0.01
Commun. Prob.
max
min
B
SPP1 − CD44
PTN − SDC4
PTN − PTPRZ1
PTN − ALK
PSAP − GPR37
NRG3 − ERBB4
NRG1 − ERBB4
NAMPT − INSR
FGF1 − FGFR3
FGF1 − FGFR2
Oligodendrocytes −> Oligodendrocytes
Oligodendrocytes −> Astrocytes
Oligodendrocytes −> T cells
Astrocytes −> Oligodendrocytes
Astrocytes −> Astrocytes
Astrocytes −> Microglias
Astrocytes −> Oligodendrocyte precursor cells
Astrocytes −> Pericytes
Astrocytes −> Neurons
Astrocytes −> T cells
Microglias −> Oligodendrocytes
Microglias −> Astrocytes
Microglias −> T cells
Oligodendrocyte precursor cells −> Oligodendrocytes
Oligodendrocyte precursor cells −> Astrocytes
Oligodendrocyte precursor cells −> Microglias
Oligodendrocyte precursor cells −> Oligodendrocyte precursor cells
Oligodendrocyte precursor cells −> Pericytes
Oligodendrocyte precursor cells −> Neurons
Oligodendrocyte precursor cells −> T cells
Endothelial cells −> Oligodendrocytes
Endothelial cells −> Astrocytes
Endothelial cells −> Oligodendrocyte precursor cells
Endothelial cells −> Endothelial cells
Endothelial cells −> Pericytes
Endothelial cells −> Fibroblast−like cells
Endothelial cells −> T cells
Pericytes −> Oligodendrocytes
Pericytes −> Astrocytes
Pericytes −> Oligodendrocyte precursor cells
Pericytes −> T cells
Neurons −> Oligodendrocytes
Neurons −> Astrocytes
Neurons −> Microglias
Neurons −> Oligodendrocyte precursor cells
Neurons −> Pericytes
Neurons −> Neurons
Neurons −> T cells
Fibroblast−like cells −> Oligodendrocytes
Fibroblast−like cells −> Astrocytes
Fibroblast−like cells −> T cells
T cells −> Oligodendrocytes
T cells −> Astrocytes
T cells −> T cells
p−value
p < 0.01
Commun. Prob.
max
min
