# Supplementary material for: MAFF as a potential novel protective factor against Parkinson’s disease: evidence from Mendelian randomization and single-cell RNA sequencing
Source: Front Aging Neurosci. 2026 Sep 11;18:1759121. doi: 10.3389/fnagi.2026.1759121 (PMC13612465; doi:10.3389/fnagi.2026.1759121)
Supplement: Supplementary file 3 [file Data_Sheet_3.PDF]

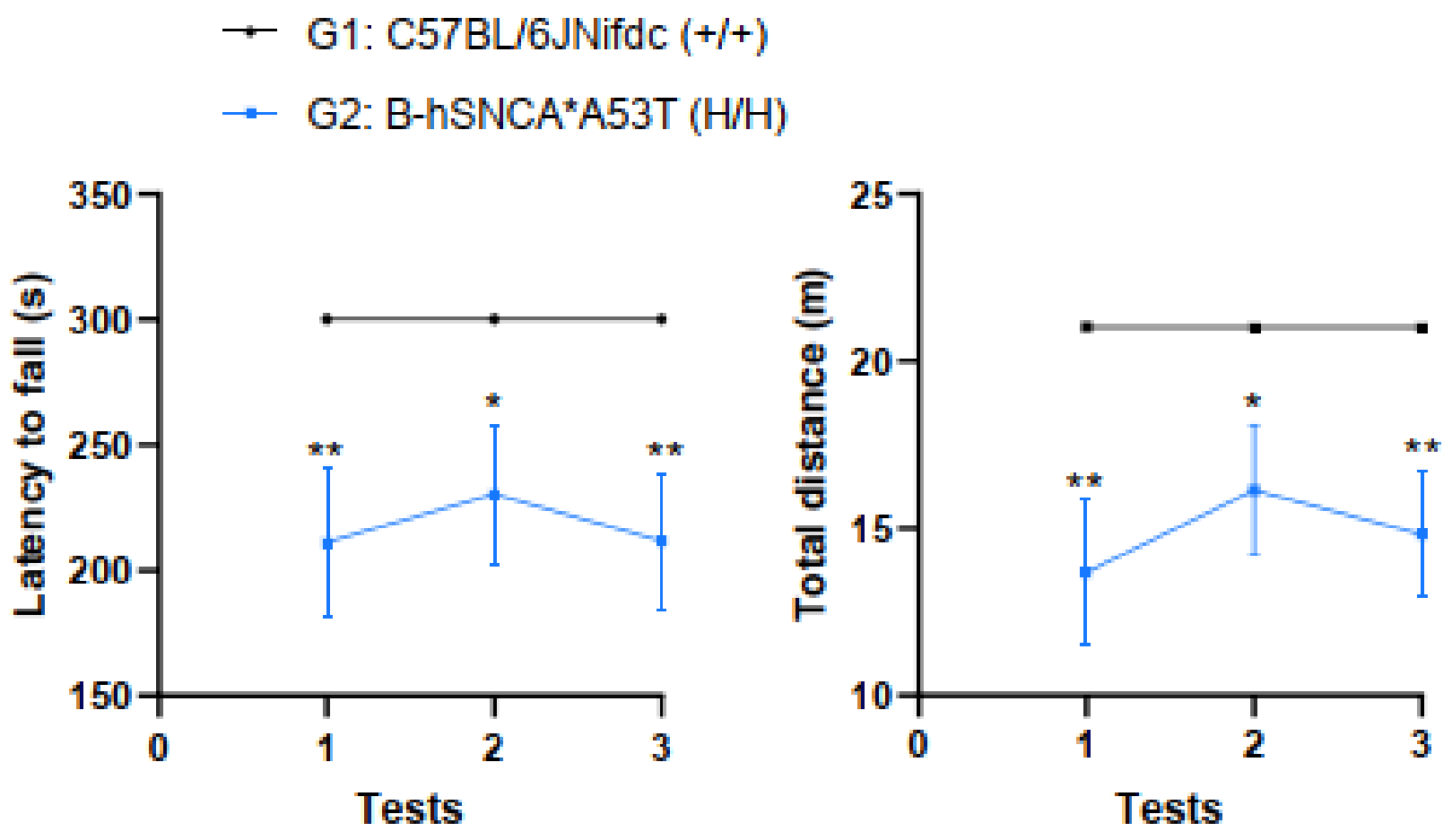

Behavioral performance in wild-type C57BL/6JNifdc and homozygous B-hSNCA*A53T mice. Rotarod tests were conducted to assay the behavioral performance in wild-type C57BL/6JNifdc and homozygous B-hSNCA*A53T mice (n=8 for per sex, 6-7-week-old). Rotarod tests were performed to assay the motor coordination. The latency to fall and total distance were significantly decreased in homozygous B-hSNCA*A53T mice at all the three tests, showing the impairment of motor coordination and balance. Values are expressed as mean ± SEM. At each test point significance was determined by unpaired t test. *P < 0.05, **P < 0.01, ***P < 0.001.
