# Supplementary material for: MAFF as a potential novel protective factor against Parkinson’s disease: evidence from Mendelian randomization and single-cell RNA sequencing
Source: Front Aging Neurosci. 2026 Sep 11;18:1759121. doi: 10.3389/fnagi.2026.1759121 (PMC13612465; doi:10.3389/fnagi.2026.1759121)
Supplement: Supplementary file 4 [file Table_1.docx]

| **Primer** | **Sequence** | |
| --- | --- | --- |
| MAFF F | GATCTAGCCACATGGGGTCCT | |
| MAFF R | GCTTGACCTTCAGGGCTTTG |  |
| Internal reference -GAPDH F | TGTGTCCGTCGTGGATCTGA |  |
| Internal reference -GAPDH R | GAGTTGCTGTTGAAGTCGCA |  |

**The primer sequence of RT-qPCR analysis in mouse samples**
