# Supplementary material for: MAFF as a potential novel protective factor against Parkinson’s disease: evidence from Mendelian randomization and single-cell RNA sequencing
Source: Front Aging Neurosci. 2026 Sep 11;18:1759121. doi: 10.3389/fnagi.2026.1759121 (PMC13612465; doi:10.3389/fnagi.2026.1759121)
Supplement: Supplementary file 6 [file Table_5.docx]

**Supplementary Table S5**. The results of the reverse MR analysis.

| id.exposure | id.outcome | method | nsnp | b | se | pval | Q_pval | Egger_pval |
| --- | --- | --- | --- | --- | --- | --- | --- | --- |
| ebi-a-GCST90018894 | eqtl-a-ENSG00000185022 | MR Egger | 3 | 0.0204 | 0.1558 | 0.9171 | 0.4177 | - |
|  |  | IVW | 3 | 0.0347 | 0.0351 | 0.3233 | 0.7169 | 0.9402 |
|  |  | Weighted median | 3 | 0.0371 | 0.0404 | 0.3584 | - | - |
|  |  | Simple mode | 3 | 0.0520 | 0.0478 | 0.3907 | - | - |
|  |  | Weighted mode | 3 | 0.0427 | 0.0473 | 0.4618 | - | - |
