# Supplementary material for: MAFF as a potential novel protective factor against Parkinson’s disease: evidence from Mendelian randomization and single-cell RNA sequencing
Source: Front Aging Neurosci. 2026 Sep 11;18:1759121. doi: 10.3389/fnagi.2026.1759121 (PMC13612465; doi:10.3389/fnagi.2026.1759121)
Supplement: Supplementary file 7 [file Table_6.docx]

**Supplementary Table S6**. The result of the Steiger test.

| exposure | outcome | correct_causal_direction | steiger_pval |
| --- | --- | --- | --- |
| Parkinson | MAFF expression | TRUE | 0.130 |
